# IMPLEMENTATION OF THE ELDER MISTREATMENT EMERGENCY DEPARTMENT TOOLKIT AT LYNDON B. JOHNSON HOSPITAL

**DOI:** 10.1093/geroni/igac059.1593

**Published:** 2022-12-20

**Authors:** Sherry Plummer, Jason Burnett, Ruthann Froberg, Randi Campetti, Kim Dash

**Affiliations:** Lyndon B. Johnson General Hospital, Houston, Texas, United States; The University of Texas McGovern Medical School at Houston, Houston, Texas, United States; Education Development Center, Inc., Waltham, Massachusetts, United States; Education Development Center, Waltham, Massachusetts, United States; Education Development Center, Waltham, Massachusetts, United States

## Abstract

Lyndon B. Johnson Hospital (LBJ) has the busiest Level III trauma and emergency department (ED) in Texas. Located in the busiest Houston zip code for Adult Protective Services reports, LBJ staff routinely assess older adults for mistreatment with no formal screening and response protocol. An ED-wide staff assessment revealed formal training needs for elder mistreatment (EM) detection, management, and reporting. Between October and December 2019, 55% of ED bedside nurses were trained along with 75% of charge nurses, and 12 clinical and nurse case managers, resulting in improved knowledge regarding EM screening and response best practices. In January 2021, LBJ staff implemented the Elder Mistreatment Screening and Response Tool (EM-SART). This resulted in 1,218 complete screens, 23 cases of suspected EM (2%), and 4 confirmed EM cases. Despite the pandemic and other challenges, LBJ staff demonstrated resilience and dedication, and reported EM training, screening, and response protocol efficacy.

